# The relationship between sleep quality and academic achievement among students in health-related disciplines: a cross-sectional study

**DOI:** 10.1186/s12909-026-08861-0

**Published:** 2026-02-21

**Authors:** Asuman Okur, Arif Keskin, Özlem Aydın Berktaş

**Affiliations:** 1 Giresun University Faculty of Medicine Department of Public Health, Giresun, Turkey; 2 Giresun University Faculty of Medicine Department of Anatomy, Giresun, Turkey; 3 Giresun University Health Services Vocational School, Giresun, Turkey

**Keywords:** Sleep quality, Academic performance, Health sciences students, Study habits, Sleep hygiene, Medical education

## Abstract

**Background:**

Sleep plays a crucial role in maintaining both physical and cognitive functions, directly influencing academic performance among university students. This study aimed to determine the level of sleep quality and its associated personal and academic variables among students enrolled in the Faculties of Medicine, Dentistry, and Health Sciences at Giresun University.

**Methods:**

This cross-sectional study was conducted between May and June 2025 among 315 students selected through stratified sampling from a population of 850. Data were collected via an online questionnaire including sociodemographic, academic, and sleep-related variables, as well as the Pittsburgh Sleep Quality Index (PSQI). The sample size was calculated assuming a 50% prevalence, a 95% confidence level, a 5% margin of error, and an additional 15% to compensate for nonresponse. Statistical analyses included correlation tests and logistic regression.

**Results:**

Among participants, 56.8% strongly agreed and 31.4% agreed that their sleep pattern affects academic performance. According to the PSQI cut-off (> 5), 255 participants (80.9%) had poor sleep quality, while 60 (19.1%) had good sleep quality. A weak positive correlation was found between sleep duration on the exam day and most recent exam grade (*r* = 0.122, *p* = 0.030). GPA showed a weak positive correlation with sleep duration on the exam day (*r* = 0.166, *p* = 0.003), though not with total PSQI score. Dentistry students had significantly higher odds of poor sleep quality compared with both midwifery/nursing and medical students in multivariable analysis. Each additional hour of study on the exam day increased the odds of PSQI based poor sleep quality by 10% (OR = 1.10, *p* = 0.045).

**Conclusions:**

Poor sleep quality was prevalent among health-related faculty students and was significantly associated with study habits. Interventions that promote effective study planning, adequate rest, and awareness of sleep hygiene are essential to improve academic performance and overall well-being, as well as to prepare students for future professional demands involving shift work and on-call duties.

**Supplementary Information:**

The online version contains supplementary material available at 10.1186/s12909-026-08861-0.

## Introduction

Sleep constitutes a fundamental component of the circadian rhythm and is essential for maintaining physiological and psychological homeostasis. It not only enables physical and mental restoration but also plays a crucial role in supporting learning, memory, and cognitive performance through the regulation of neuronal plasticity and detoxification processes within brain cells. These cellular repair mechanisms are instrumental in preventing neurodegenerative processes and maintaining overall brain health [1, 2].

Accordingly, sleep is indispensable not only for sustaining cognitive functions but also for preserving the proper functioning of multiple physiological systems. A growing body of evidence indicates that insufficient sleep duration increases susceptibility to psychological, neurological, and systemic disorders. Previous studies have shown that sleep deprivation contributes to a higher risk of metabolic conditions such as obesity, as well as cardiovascular abnormalities including arrhythmias and coronary artery disease [3–6].

Adequate and high-quality sleep underpins cognitive processes that are directly associated with academic performance, including attention, learning capacity, memory consolidation, and emotional regulation [7]. Recent research has consistently demonstrated the detrimental impact of inadequate sleep on university students’ academic outcomes [7, 8]. Taken together, these findings highlight the bidirectional and multifaceted relationship between sleep and various physiological, psychological, and social processes. The interdependence of these mechanisms is both complex and vital for maintaining overall health. Hence, regular, sufficient, and high-quality sleep may be regarded as both an indicator and determinant of well-being [5].

Nevertheless, studies focusing on university students in health-related fields have revealed that even this population—expected to have a heightened awareness of health-promoting behaviors—frequently experiences insufficient sleep. In particular, students enrolled in faculties of medicine, dentistry, and nursing have been reported to exhibit poor sleep quality [5, 9].

According to the *Health Statistics Yearbook 2023*, the potential capacity of the healthcare workforce is estimated at 39.4% [10]. Within this context, investigating the relationship between sleep quality and academic performance among health sciences students holds significant importance. Understanding this association is critical not only for fostering the development of future healthcare professionals capable of delivering high-quality services but also for preparing them to cope with occupational challenges such as shift work and night duties—factors known to adversely affect sleep. Furthermore, enhancing students’ awareness regarding the importance of sleep quality may serve as a preventive measure to mitigate such risks.

Therefore the present study was designed to assess sleep quality among students enrolled in the faculties of medicine, dentistry, and health sciences (Departments of midwifery and nursing), and to identify personal and academic factors associated with sleep quality. We anticipate that the findings will contribute to the existing body of knowledge and provide valuable insights into the overall quality and resilience of the future healthcare workforce.

## Materials and methods

This cross-sectional study was conducted among students enrolled in the Faculties of Medicine, Dentistry, and Health Sciences (Departments of Midwifery and Nursing) at Giresun University. Data were collected through an online questionnaire administered between May and June 2025. The study population consisted of 850 students from these faculties.

The sample size was calculated using the formula for populations with a known universe. Assuming a prevalence of 50%, a 95% confidence level, a 5% margin of error, and an additional 15% to account for potential nonresponse, the required sample size was determined to be 305 participants. A stratified sampling method was employed based on faculty type. Although data were initially collected from 320 students, the responses of five participants were excluded due to inappropriate or incomplete data entry. Consequently, the final analysis included 315 students, resulting in a response rate of 98.4%.

Students enrolled in the Faculties of Medicine, Dentistry, and Health Sciences (Departments of Midwifery and Nursing) at Giresun University were included in the study. Participants who voluntarily agreed to take part and provided complete data were eligible for inclusion. No additional exclusion criteria were applied.

The data collection tool used in this study included two parts (see Supplementary File 1–2): the first part was a questionnaire developed by the researchers, consisting of 44 questions, and the second part was the Pittsburgh Sleep Quality Index (PSQI). The PSQI is a self-reported scale designed to assess sleep quality and sleep disturbances over the previous month. PSQI originally developed by Buysse et al. in 1989 [11], the Turkish validity and reliability study was conducted by Ağargün, Kara, and Anlar in 1996, confirming its suitability for the Turkish population [12]. The Cronbach’s alpha coefficient for internal consistency was reported as 0.80.

The PSQI comprises seven components: subjective sleep quality, sleep latency, sleep duration, habitual sleep efficiency, sleep disturbances, use of sleep medication, and daytime dysfunction. Each component is scored on a scale from 0 to 3, and the total PSQI score—ranging from 0 to 21—is obtained by summing the component scores. Higher scores indicate poorer sleep quality. A total PSQI score of ≤ 5 indicates “good sleep quality,” while a score > 5 reflects “poor sleep quality,” as originally defined in the PSQI validation study by Buysse et al. [11]. Classification of sleep quality (poor/good) was based on the PSQI.

Academic achievement was assessed using students’ grade point averages (GPA) and most recent exam scores. GPA and most recent exam scores were self-reported by participants via the online questionnaire. Demographic and personal variables included age, gender, height, weight, body mass index (BMI), and last exam scores. Sleep medication use was assessed by asking the question, “Do you use any medication to help you sleep?” (Yes/No).

Descriptive statistics were presented as numbers, percentages, means, medians, and standard deviations. The normality of data distribution was evaluated using the Kolmogorov–Smirnov test, histogram inspection, and skewness-kurtosis values. For group comparisons, independent samples t-tests and ANOVA were applied. Associations between categorical variables were analyzed using the chi-square test. Since all numerical variables showed a normal distribution, the relationships between them were evaluated using the Pearson correlation test.

Multivariable logistic regression (backward Wald) analysis was performed to identify factors independently associated with poor sleep quality. PSQI-based poor sleep quality (PSQI score > 5) was included as the dependent variable. The independent variables entered into the model were age, gender, academic department (midwifery/nursing, dentistry, medicine), presence of a self-reported psychiatric disorder, time required to feel rested after waking (≤ 30 min, > 30 min), frequency of night awakenings (0–1 times, ≥ 2 times per night), presence of a tablet in the bedroom, study time on the day before the most recent exam (hours), and most recent exam grade. Categorical variables were included in the model using indicator (dummy) variables with the following reference categories: male gender, midwifery/nursing department, absence of psychiatric disorder, feeling rested within ≤ 30 min, 0–1 night awakenings, and absence of a tablet in the bedroom. Prior to the logistic regression analysis, multicollinearity among independent variables was assessed using variance inflation factor (VIF) and tolerance statistics. Results were presented as regression coefficients (B), odds ratios (OR) with 95% confidence intervals, Wald statistics, and p values.

Statistical analyses were conducted using IBM SPSS Statistics for Windows, version 25.0 (IBM Corp., Armonk, NY, USA). A p-value of < 0.05 was considered statistically significant.

## Results

Among the participants, 38.4% were enrolled in the faculty of dentistry, 30.5% in the faculty of medicine, 16.8% in the department of nursing, and 14.3% in the department of midwifery. Regarding the distribution by academic year, 33.3% were first-year students, 27.9% were in the second year, 20.0% were in the third year, and 18.7% were in the fourth year.

The majority of participants were female (73.7%), while 26.3% were male. Only 4.4% of the students reported having a paid job. The proportion of students who consumed alcohol was 16.5%, and 18.7% reported smoking. A diagnosed psychiatric disorder was reported by 7.0% of participants, while 3.8% stated that they used medication to help them sleep. Among the participants, 56.8% strongly agreed and 31.4% agreed that their sleep pattern affects their academic performance, while 10.5% were neutral and 1.3% disagreed. When asked whether they noticed any change in their academic performance during periods of sleep disturbance, 85.7% reported a decrease in performance, 1.6% reported an increase, and 12.7% stated that it did not change. Regarding perceived sleep quality, 25.1% of the students rated their sleep as good, 54.3% as moderate, and 20.6% as poor (Table 1).

**Table 1:** Demographic Characteristics of the Participants

	*N*	%
Department		
Dentistry	121	38.4
Midwifery	45	14.3
Nursing	53	16.8
Medicine	96	30.5
Year of Study		
1 st	105	33.3
2 nd	88	27.9
3 rd	63	20.0
4 th	59	18.7
Gender		
Male	83	26.3
Female	232	73.7
Employment Status		
Employed	14	4.4
Unemployed	301	95.6
Alcohol Use		
Yes	52	16.5
No	263	83.5
Smoking Status		
Smoker	59	18.7
Non-smoker	256	81.3
Presence of Psychiatric Disorder		
Yes	22	7.0
No	293	93.0
Use of Medication to Sleep		
Yes	12	3.8
No	303	96.2
Do you think your sleep pattern affects your academic performance?		
Strongly agree	179	56.8
Agree	99	31.4
Neutral	33	10.5
Disagree	4	1.3
Do you notice any change in your academic performance during periods of sleep disturbance?		
Yes, my performance increases	5	1.6
Yes, my performance decreases	270	85.7
No, it does not change	40	12.7
Perceived Sleep Quality		
Good	79	25.1
Moderate	171	54.3
Poor	65	20.6

**Table 2:** Correlation of Sleep Score with Academic Performance and Sleep Characteristics

		Average Daily Study Time During Exam Week (hours)	Study Time on the Day Before the Exam (hours)	Most Recent Exam Grade	Cumulative Grade Point Average (GPA)	Sleep quality score (PSQI)
Average daily sleep duration during exam week	r	-0.139	-0.073	0.058	-0.067	-0.030
	p	0.013	0.199	0.307	0.241	0.598
Sleep duration on the day of the exam	r	-0.279	-0.444	0.122	0.166	-0.249
	p	0.001	0.001	0.030	0.003	0.001
Sleep quality score (PSQI)	r	0.127	0.114	-0.123	-0.050	-
	p	0.024	0.042	0.029	0.382	-
Most recent exam grade	r	-0.108	-0.134	-	-	-
	p	0.055	0.018	-	-	-
Cumulative grade point average (GPA)	r	0.047	0.093	-	-	-
	p	0.410	0.100	-	-	-

A significant negative correlation was observed between average daily study time during exam week and average daily sleep duration during exam week (*r*= − 0.139, *p* = 0.013). No significant correlation was found between study time on the day before the exam and average daily sleep duration during exam week (*r*= − 0.073, *p* = 0.199). Similarly, most recent exam grade was not significantly correlated with average daily sleep duration during exam week (*r* = 0.058, *p* = 0.307).

A weak negative correlation was observed between sleep duration on the exam day and average daily study time during exam week (*r*= − 0.279, *p* < 0.001). Additionally, a moderate negative correlation was found between sleep duration on the exam day and study time on the day before the exam (*r*= − 0.444, *p* < 0.001). A weak positive and significant correlation was also observed between sleep duration on the exam day and most recent exam grade (*r* = 0.122, *p* = 0.030). Overall, sleep quality score (PSQI) was significantly associated with average daily study time during exam week, study time on the day before the exam, and most recent exam grade.

Regarding cumulative academic performance, a weak positive correlation was observed between GPA and sleep duration on the exam day (*r* = 0.166, *p* = 0.003). However, GPA was not significantly correlated with total PSQI score or average daily sleep duration during exam week (Table 2).

**Table 3:** Distribution of Academic Variables According to PSQI-Based Sleep Quality

Variable	Poor Sleep Quality^1^ (*n* = 255) (Mean ± SD)	Good Sleep Quality^2^ (*n* = 60) (Mean ± SD)	t (313)	*p*
Cumulative GPA	72.1 ± 10.6	73.7 ± 13.1	1.005	0.316
Most Recent Exam Grade	71.1 ± 14.5	75.3 ± 14.0	2.038	0.042
Screen Time (hours)	5.9 ± 2.5	5.7 ± 2.1	–0.576	0.565
Sleep Duration on the day of the Exam (hours)	4.5 ± 1.8	5.3 ± 1.6	2.920	0.004
Average Daily Sleep Time During Exam Week (hours)	6.3 ± 4.7	5.8 ± 3.4	0.886	0.376
Study Time on the Day Before Exam (hours)	8.0 ± 3.5	6.5 ± 3.1	–2.899	0.004
Average Daily Study Time During Exam Week (hours)	7.1 ± 2.7	6.2 ± 2.2	–2.242	0.026

^1^PSQI score > 5, ^2^PSQI score ≤ 5

A significant association was observed between study time on the day before the exam and sleep quality. Students with poor sleep quality reported studying 8.0 ± 3.5 h on the day before the exam, whereas those with good sleep quality studied 6.5 ± 3.1 h, with the difference reaching statistical significance (*p* = 0.004).

When comparing sleep quality with academic performance and certain habits, cumulative GPA did not differ significantly between groups: students with poor sleep quality had a GPA of 72.1 ± 10.6, while those with good sleep quality had a GPA of 73.7 ± 13.1 (*p* = 0.316). In contrast, the most recent exam grade was significantly higher in students with good sleep quality (75.3 ± 14.0) compared to those with poor sleep quality (71.1 ± 14.5; *p* = 0.042).

No significant differences were observed between groups regarding screen time (5.9 ± 2.5 vs. 5.7 ± 2.1 h; *p* = 0.565). However, sleep duration on the exam day was significantly shorter in students with poor sleep quality (4.5 ± 1.8 h) compared to those with good sleep quality (5.3 ± 1.6 h; *p* = 0.004). Furthermore, average daily study time during exam week was higher in students with poor sleep quality (7.1 ± 2.7 h) than in those with good sleep quality (6.2 ± 2.2 h), and this difference was statistically significant (*p* = 0.026) (Table 3).

**Table 4:** Relationship Between PSQI-Based Sleep Quality and Lifestyle Variables

Variable	Poor Sleep Quality^1^ *n* (%)	Good Sleep Quality ^2^ *n* (%)	χ²	*p*
Gender			0.150	0.698
Male	66 (79.5)	17 (20.5)		
Female	189 (81.5)	43 (18.5)		
Department			12.403	0.002
Dentistry	105 (86.8)	16 (13.2)		
Midwifery/Nursing	68 (69.4)	30 (30.6)		
Medicine	82 (85.4)	14 (14.6)		
Alcohol Consumption			2.278	0.131
Yes	46 (88.5)	6 (11.5)		
No	209 (79.5)	54 (20.5)		
Smoking Status			0.008	0.930
Smoker	48 (81.4)	11 (18.6)		
Non-smoker	207 (80.9)	49 (19.1)		
Diagnosed Psychiatric Disorder			3.226	0.072
Yes	21 (95.5)	1 (4.5)		
No	234 (79.9)	59 (20.1)		
Use of Medication to Sleep			2.935	0.132
Yes	12 (100.0)	0 (0.0)		
No	243 (80.2)	60 (19.8)		
Chronic Disease			1.038	0.308
Yes	16 (72.7)	6 (27.3)		
No	239 (81.6)	54 (18.4)		
Presence of Phone in Bedroom			0.022	0.881
Yes	250 (80.9)	59 (19.1)		
No	5 (83.3)	1 (16.7)		
Presence of Tablet in Bedroom			9.822	0.002
Yes	174 (86.1)	28 (13.9)		
No	81 (71.7)	32 (28.3)		
Presence of TV in Bedroom			1.135	0.287
Yes	26 (74.3)	9 (25.7)		
No	229 (81.8)	51 (18.2)		
Presence of Computer in Bedroom			0.002	0.965
Yes	80 (80.8)	19 (19.2)		
No	175 (81.0)	41 (19.0)		
Tea/Caffeine Consumption			2.111	0.348
None	17 (73.9)	6 (26.1)		
1–2 cups	176 (80.0)	44 (20.0)		
> 2 cups	62 (86.1)	10 (13.9)		
Lighting Level in Bedroom			1.092	0.579
Dim	73 (83.0)	15 (17.0)		
Bright	3 (100.0)	0 (0.0)		
Dark	179 (79.9)	45 (20.1)		
Time to Feel Rested After Waking			6.792	0.009
≤ 30 min	140 (76.1)	44 (23.9)		
> 30 min	115 (87.8)	16 (12.2)		
Number of Night Awakenings			6.531	0.011
0	110 (76.9)	33 (23.1)		
1	72 (79.1)	19 (20.9)		
2	37 (86.0)	6 (14.0)		
3	36 (94.7)	2 (5.3)		
Total	255 (80.9)	60 (19.1)		

^1^PSQI score > 5, ^2^PSQI score ≤ 5

According to the PSQI cut-off value (> 5), 255 participants (80.9%) were classified as having poor sleep quality, while 60 (19.1%) had good sleep quality. The associations between sleep quality and sociodemographic and lifestyle variables were examined. No significant differences were observed in sleep quality according to gender (*p* = 0.698). Similarly, alcohol consumption (*p* = 0.131), smoking status (*p* = 0.930), presence of a diagnosed psychiatric disorder (*p* = 0.072), use of medication to sleep (*p* = 0.132), presence of a chronic disease (*p* = 0.308), presence of a phone in the bedroom (*p* = 0.881), presence of a television in the bedroom (*p* = 0.287), or presence of a computer in the bedroom (*p* = 0.965) were not significantly associated with sleep quality. Tea/caffeine consumption also did not differ significantly between groups (*p* = 0.348).

A significant association was found between department and sleep quality (*p* = 0.002). Poor sleep quality was more prevalent among students in dentistry (86.8%) and medicine (85.4%), whereas the prevalence was lower in midwifery/nursing students (69.4%). Additionally, the presence of a tablet in the bedroom was significantly associated with poor sleep quality; 86.1% of students with a tablet had poor sleep quality, compared to 71.7% of students without a tablet (*p* = 0.002).

Regarding sleep recovery after awakening, 76.1% of students who felt rested within 30 min had poor sleep quality, whereas 87.8% of those who required more than 30 min to feel rested exhibited poor sleep quality (*p* = 0.009). The frequency of night awakenings was positively associated with poor sleep quality, increasing from 76.9% among students who did not wake up at night to 94.7% among those who awoke three times (*p* = 0.011).

No significant differences in sleep quality were observed according to bedroom lighting conditions (*p* = 0.300), with the majority of participants (71.1%) reporting sleeping in dark conditions (Table 4).

**Table 5:** Primary Independent Variables Associated with PSQI-Based Poor Sleep Quality

Variable	B	Wald	*p*	Odds Ratio (OR)	95% CI
Age	-0.033	0.253	0.615	0.97	0.85–1.10
Gender					
Male	Ref	Ref	Ref	Ref	Ref
Female	0.80	4.734	0.030	2.24	1.08–4.61
Department					
Midwifery/Nursing	Ref	Ref	Ref	Ref	Ref
Dentistry	0.95	4.99	0.025	2.58	1.12–5.93
Medicine	-0.17	0.13	0.721	0.84	0.33–2.17
Presence of Psychiatric Disorder					
No	Ref	Ref	Ref	Ref	Ref
Yes	0.64	1.57	0.210	1.90	0.70–5.18
Time to Feel Rested After Waking					
≤30 min	Ref	Ref	Ref	Ref	Ref
>30 min	0.86	9.01	0.003	2.37	1.35–4.15
Night Awakenings					
0–1 times	Ref	Ref	Ref	Ref	Ref
≥2 times	0.68	5.10	0.024	2.00	1.10–3.55
Presence of Tablet in Bedroom					
No	Ref	Ref	Ref	Ref	Ref
Yes	-0.58	0.28	0.868	0.56	0.48–1.88
Study Time on the Day Before Exam (hours)	0.09	2.97	0.045	1.10	1.01–1.44
Most Recent Exam Grade	–0.01	1.15	0.285	0.99	0.97–1.01

VIF_all_ < 2.19; tolerance = 0.46–0.99

Covariates: Age, gender, academic department, self-reported psychiatric disorder, time to feel rested after waking, frequency of night awakenings, presence of a tablet in the bedroom, study time before the last exam, and most recent exam grade

Logistic regression analysis revealed significant differences in sleep quality according to several demographic, academic, and sleep-related characteristics. Female students had significantly higher odds of poor sleep quality compared with male students (OR = 2.24). Students in dentistry were 2.58 times more likely to experience poor sleep quality compared to those in midwifery/nursing. Students who required more than 30 min to feel rested after waking had a 2.37-fold higher risk of poor sleep quality compared to those who felt rested within 30 min (OR = 2.37, *p* = 0.003).

Although students with a history of psychiatric disorder had a 1.90-fold higher likelihood of poor sleep quality compared to those without such a history, this difference did not reach statistical significance (OR = 1.90, *p* = 0.210). The likelihood of poor sleep quality was 2.00 times higher among students who awoke two or more times during the night (OR = 2.00, *p* = 0.024). Finally, each additional hour of study on the day before the exam was associated with a 10% increase in the odds of poor sleep quality (OR = 1.10, *p* = 0.045) (Table 5). Additional analysis using medicine as the reference category showed that dentistry students had significantly higher odds of poor sleep quality compared with medical students (OR = 2.91, 95% CI: 1.44–5.88, *p* = 0.003).

## Discussion

This study investigated the relationship between sleep quality and academic performance among students enrolled in health-related faculties at Giresun University. Consistent with the literature, assessment using the PSQI showed that only one-quarter of the participants had good sleep quality, whereas the vast majority (80.9%) were classified as having poor sleep quality. Previous studies have reported prevalence rates ranging from 60% to 90% [9, 13]. Most participants indicated that their sleep patterns affected their academic performance. Suardiaz-Muro et al. reported that university students experience insufficient and low-quality sleep during exam periods, which negatively affects their academic performance, and students believe that more sleep would enhance their performance [13].

Our data revealed a weak negative correlation between average study time and sleep duration during the exam week; however, this negative correlation increased to a moderate level on the exam day. This finding suggests that sleep duration decreases as study time increases toward the exam day. Moreover, a weak positive association was observed between study duration during the exam week and exam day and sleep scores, indicating that increased study time is associated with poorer sleep quality. A weak negative correlation was also found between sleep score and the most recent exam grade, suggesting that lower sleep quality may be linked to lower academic performance. A weak positive and significant correlation between sleep duration on the exam day and overall GPA further indicates that students who sleep longer on exam day may achieve higher academic outcomes. These findings are consistent with previous studies in the literature.

The negative association between exam day study time and sleep quality suggests that students compromise sleep to accommodate more study hours. While increased study time was associated with poorer sleep quality, and poorer sleep quality was also associated with lower most recent exam grades. Previous research has reported mixed results regarding the relationship between sleep duration, quality, and academic achievement, with some studies finding no association between academic performance and sleep patterns, total sleep duration, or sleep-wake schedules [9, 14]. Sleep-related conditions such as insomnia, mood disorders, narcolepsy, and circadian rhythm disorders have been linked to lower academic performance and are commonly observed among medical students [15]. Cvejic et al. reported that one-quarter of medical students sleep less than 7 h. Earlier bedtimes, regular sleep-wake schedules, and shorter but less restorative sleep were associated with better psychological well-being and higher academic performance [16]. Conversely, Aydın and Aydın found a positive relationship between academic self-efficacy and long-term sleep quality [17].

Although some studies, such as those from Indonesia, found no relationship between sleep duration and academic achievement, they reported associations between wake-up times and academic success [18]. Carter et al., in a meta-analysis, found that media device access and use at bedtime were significantly associated with insufficient sleep duration, poor sleep quality, and excessive daytime sleepiness [19]. While the presence of a tablet in the bedroom was associated with poor sleep quality in univariate analysis, this association did not persist after adjustment for potential confounders in the multivariable model. However, this association should be interpreted cautiously, given that tablet use was assessed in a limited and non-quantitative manner.

The widespread use of tablets among higher education students may reflect the substitution of traditional study materials such as printed textbooks, notebooks, and desktop/laptop computers.

After controlling for demographic, academic, and sleep-related factors, dentistry students had significantly higher odds of poor sleep quality compared with both midwifery/nursing and medical students. This finding may be related to certain characteristics of dental education that differ from medical and midwifery/nursing training. Dental curricula typically involve intensive practical skills training, laboratory work, and early patient-based clinical practice. These activities often require prolonged fixed postures, fine motor precision, and sustained attention, and clinical performance is evaluated continuously and individually. Such demands may act as ongoing stressors and potentially contribute to poorer sleep quality. Previous studies have also reported poor sleep quality among dental students and suggested that this may be associated with academic demands and stress [20–22].

Additionally, the frequency of night awakenings and longer times to feel rested in the morning were associated with poorer sleep quality. A positive and significant correlation was observed between time to feel rested and sleep scores, indicating that delays in feeling rested are accompanied by decreased sleep quality.

Limitations of this study include its cross-sectional design, which precludes causal inferences, and the timing of the survey during the final exam period, limiting the generalizability of sleep quality findings to the entire academic year. Not all academic levels were included, and non-probability sampling may restrict external validity. Also academic performance indicators were self-reported and may be subject to recall or reporting bias.

Although the use of sleep medications was assessed, detailed information regarding the type and dosage of these medications was not collected. While the prevalence of sleep medication use in the present study was low (3.8%) and no significant association with sleep quality was observed, the lack of detailed medication data may have limited our ability to fully account for their potential effects on sleep quality and academic performance. As the study was conducted in a single university, the findings may not be generalizable to all health science students in different regions or educational systems.

The strengths of this study include stratified sampling according to health-related faculties and the use of the validated Pittsburgh Sleep Quality Index. Additionally, conducting the survey during the final exam period allowed for assessment of sleep-academic performance relationships under peak exam stress and enabled comparisons across different health faculties.

There is a need for multicenter studies with larger sample sizes to assess sleep quality among students enrolled in higher education institutions in the health sciences in Turkey. Well-designed prospective studies with a longitudinal follow-up may help to clarify the causal relationship between sleep quality and academic achievement. In addition, future research would benefit from assessing sleep quality at different time points throughout the academic year, rather than focusing solely on examination periods, in order to enhance the generalizability of the findings.

## Conclusion

Overall, students in health-related faculties exhibited generally poor sleep quality. The findings indicate significant associations between sleep quality and study-related behaviors. These results highlight the potential importance of considering sleep in discussions of student well-being and academic functioning, particularly during examination periods. Interventions aimed at promoting healthy study routines and sleep hygiene may be beneficial, but prospective and interventional studies are needed to determine their effects on academic outcomes and future professional adaptation.

## Supplementary Information

Supplementary Material 1.

Supplementary Material 2.

## Data Availability

The datasets used and/or analysed during the current study are available from the corresponding author on reasonable request.

## Abbreviations

GPA: Grade Point Average
IBM: International Business Machines Corporation
OR: Odds Ratio
USA: United States of America
PSQI: Pittsburgh Sleep Quality Index
SD: Standard Deviation
SPSS: Statistical Package for the Social Sciences
