# Supplementary material for: The relationship between sleep quality and academic achievement among students in health-related disciplines: a cross-sectional study
Source: BMC Med Educ. 2026 Feb 21;26:445. doi: 10.1186/s12909-026-08861-0 (PMC13001357; doi:10.1186/s12909-026-08861-0)
Supplement: Supplementary file 1 — Supplementary Material 1. [file 12909_2026_8861_MOESM1_ESM.pdf]

PITTSBURGH SLEEP QUALITY INDEX (PSQI)

Please answer the following questions considering your sleep habits during the past month only.		
1.	During the past month, when have you usually gone to bed at night?	
2.	During the past month, how long (in minutes) has it usually taken you to fall asleep each night?	
3	During the past month, when have you usually gotten up in the morning?	
4.	During the past month, how many hours of actual sleep did you get at night? (This may be different from the number of hours you spent in bed.)	

During the past month, how often have you had trouble sleeping because you...

		Not during the past month	Less than once a week	Once or twice a week	Three or more times a week
5a	Cannot get to sleep within 30 minutes				
5b	Wake up in the middle of the night or early morning				
5c	Have to get up to use the bathroom				
5d	Cannot breathe comfortably				
5e	Cough or snore loudly				
5f	Feel too cold				
5g	Feel too hot				
5h	Have bad dreams				
5i	Have pain				
5j	Other reasons (please describe): ___________				

		Not during the past month	Less than once a week	Once or twice a week	Three or more times a week
6	During the past month, how often have you taken medicine (prescribed or "over the counter") to help you sleep?				
7	During the past month, how often have you had trouble staying awake while driving, eating meals, or engaging in social activity?				
8	During the past month, how much of a problem has it been for you to keep up enough enthusiasm to get things done?				

9. During the past month, how would you rate your overall sleep quality?

☐ Very good
☐ Fairly good
☐ Fairly bad
☐ Very bad
