# Supplementary material for: The relationship between sleep quality and academic achievement among students in health-related disciplines: a cross-sectional study
Source: BMC Med Educ. 2026 Feb 21;26:445. doi: 10.1186/s12909-026-08861-0 (PMC13001357; doi:10.1186/s12909-026-08861-0)
Supplement: Supplementary file 2 — Supplementary Material 2. [file 12909_2026_8861_MOESM2_ESM.pdf]

# Sociodemographic Characteristics and Descriptive Information Form

1. **Age:**
2. **Gender:**
    1. Male
    2. Female
3. **Department:**
    1. Nursing
    2. Midwifery
    3. Dentistry
    4. Medicine
4. **Year of study:** ………….
5. **Current living arrangement:**
    1. With family
    2. In a private dormitory
    3. In a state dormitory
    4. Living alone
    5. In a shared student house with friends
6. **Is your father currently employed?**
    1. Yes → Occupation: ……………
    2. No
7. **Is your mother currently employed?**
    1. Yes → Occupation: ……………
    2. No
8. **How would you describe your family's income level?**
    1. Low
    2. Moderate
    3. High
9. **What type of family do you have?**
    1. Nuclear family
    2. Extended family
    3. Divorced/separated family
10. **Do you work in a paid job outside of school?**
    1. Yes
    2. No
11. **Do you consume alcohol?**
    1. Yes
    2. No
12. **Do you smoke cigarettes?**
    1. Yes
    2. No
13. **Do you have any diagnosed psychiatric disorder?**
    1. Yes
    2. No
14. **Do you have any chronic illness?**
    1. Yes
    2. No
15. **Do you use any medication to help you sleep?**
    1. Yes
    2. No

16. **Do you have a phone in your bedroom?**
    1. Yes
    2. No
17. **Do you have a tablet device in your bedroom?**
    1. Yes
    2. No
18. **Do you have a TV in your bedroom?**
    1. Yes
    2. No
19. **Do you have a computer in your bedroom?**
    1. Yes
    2. No
20. **How much time do you spend daily in front of a screen?**..................... hours
21. **What do you usually do in the last 30 minutes before sleep? (Please mark only one)**
    1. Read a book
    2. Watch television
    3. Listen to music
    4. Use or talk on the phone
    5. Study
    6. Other: ……………
22. **Do you fall asleep immediately after going to bed?**
    1. Yes
    2. No
23. **How often do you consume tea, coffee, or other caffeinated beverages?**
    1. Never
    2. Once a day
    3. Twice a day
    4. Three or more times a day
24. **On average, how many hours do you sleep per night?**.................... hours
25. **After waking up, how long does it take for you to feel rested?**
    1. Immediately
    2. 15–30 minutes
    3. 30 minutes – 1 hour
    4. 1–2 hours
    5. More than 2 hours
26. **How long does it usually take you to fall asleep?**........................ minutes
27. **How many times do you wake up during the night?**.......................times
28. **Do you use electronic devices before sleeping?**
    1. Yes, regularly
    2. Yes, occasionally
    3. Rarely
    4. No
29. **What is the light condition in your room while sleeping?**
    1. Completely dark
    2. Dim light
    3. Bright light

30. **Do you think your sleep pattern affects your academic performance?**
    1. Strongly agree
    2. Agree
    3. Undecided
    4. Disagree
    5. Strongly disagree
31. **When your sleep pattern changes, do you notice any change in your academic performance?**
    1. Yes, it improves
    2. Yes, it decreases
    3. No, it does not change
32. **Does your sleep pattern change on weekends?**
    1. Yes, I usually sleep less
    2. Yes, I usually sleep more
    3. No, it doesn't change
33. **How would you rate your overall sleep quality?**
    1. Very poor
    2. Poor
    3. Fair
    4. Good
    5. Very good
34. **How many hours do you usually sleep during exam weeks?........................hours**
35. **How many hours do you sleep the night before an exam?............................hours**
36. **What is your study habit?**
    1. I study every day
    2. I study occasionally
    3. I study only before exams
    4. I do not study; I learn during lectures
37. **How many hours a day do you study on average?..........................hours**
38. **How many hours a day do you study during exam weeks?.....................hours**
39. **How many hours do you study the day before an exam?........................hours**
40. **How often do you have dreams?**
    1. I don't dream
    2. I dream but don't remember
    3. Occasionally
    4. Frequently
    5. I dream every day
41. **Do you have dreams during exam weeks?**
    1. No
    2. Yes
42. **How does alcohol or substance use affect your sleep?**
    1. I don't use
    2. When I use, I sleep well
    3. When I use, I sleep less
    4. When I use, I wake up rested
    5. When I use, I wake up restless/tired
    6. When I use, my sleep quality decreases
    7. When I use, I wake up feeling unwell
43. **A. What was your last exam grade? ………………………….**

B. **What is your overall grade point average?…………………….**

44. **Weight: ………………… kg**
    **Height: ………………… cm**
